# New insights into combined immunotherapy for hepatocellular carcinoma associated with liver cirrhosis

**DOI:** 10.3389/fimmu.2025.1741398

**Published:** 2025-12-18

**Authors:** Yufan Liu, Kexin Cong, Qiao Li, Dan Zhang, Jiyao Sheng

**Affiliations:** 1 Department of Hepatobiliary and Pancreatic Surgery, The Second Hospital of Jilin University, Changchun, Jilin, China; 2 Department of Surgery, The Second Hospital of Jilin University, Changchun, Jilin, China

**Keywords:** combined immunotherapy, hepatocellular carcinoma, immune microenvironment, immunotherapy, liver cirrhosis

## Abstract

Worldwide, most (80%–90%) hepatocellular carcinomas (HCCs) develop against a background of liver cirrhosis, where chronic inflammation, fibrosis, and immune dysfunction collectively shape an immunosuppressive hepatic microenvironment. Despite significant breakthroughs in HCC treatment with immune-checkpoint inhibitors, objective response rates remain limited in patients with advanced HCC, primarily because of cirrhosis-mediated remodeling of the hepatic immune microenvironment. This review systematically summarizes recent theories and mechanisms by which cirrhosis impairs immunotherapy through reshaping the hepatic immune microenvironment. It also covers alterations in the quantity, function, and metabolism of dendritic cells, T cells, macrophages, and neutrophils. Furthermore, it proposes potential intervention targets and combination therapy strategies aimed at correcting these immune abnormalities, all of which have demonstrated value in basic and translational research. In summary, cirrhosis constitutes the pathogenic foundation of HCC and represents a critical determinant of immunotherapy response. The future integration of immunotherapeutic strategies targeting the cirrhotic immune microenvironment holds promise as a key direction for enhancing immunotherapy efficacy in cirrhosis-associated HCC.

## 1 Introduction

Liver cancer ranks second among causes of cancer mortality worldwide, with roughly 866,000 new diagnoses each year (1). Hepatocellular carcinoma (HCC) is the predominant histologic type, comprising approximately 90% of primary hepatic malignancies (2). In contrast to many other common solid cancers driven primarily by stochastic, multifactorial mutational events, HCC typically emerges through a stepwise cascade of persistent hepatic injury, followed by inflammation, fibrogenesis, and ultimately cirrhosis (3). Globally, 80%–90% HCCs arise in cirrhotic livers (4, 5).

The core pathological features of liver cirrhosis include diffuse fibrosis, the formation of regenerative nodules, and significant intraliver vascular remodeling. Persistent chronic injury irreversibly alters normal lobular architecture, producing an interlaced pattern of hepatocellular parenchyma and fibrous septa (6). Activated hepatic stellate cells produce a large amount of collagen, causing excessive deposition of extracellular matrix (7). Concurrently, hepatic sinusoidal endothelial cells undergo de-fenestration and capillarization, and further elevates hepatic vascular resistance by reducing nitric oxide and increasing vasoconstrictive factors, thereby promoting the development of portal hypertension (8, 9). Cirrhosis is also accompanied by sustained infiltration of inflammatory cells and elevated expression of proinflammatory cytokines, manifesting as chronic inflammation, altered immune-cell distribution, and impaired immune function (10, 11). These alterations collectively form the histopathological basis of liver cirrhosis and provide crucial context for elucidating changes in the immune microenvironment and developing immunotherapy strategies. In liver cirrhosis, the composition and function of hepatic immune cells undergo significant alterations, manifesting as impairments in both innate and adaptive immune responses. These changes involve numerous immune cell types, including diminished dendritic cell (DC) function, T-cell shift toward an exhausted state, pro-inflammatory phenotypes in macrophages, and neutrophil-derived extracellular networks (NETs).

Current locoregional treatments, including liver resection, radiofrequency ablation, and transarterial chemoembolization (TACE), offer therapeutic benefits, but their overall efficacy diminishes considerably in patients with advanced-stage disease (12). Immunotherapy has reshaped the therapeutic landscape: blockade of the programmed cell death protein 1 (PD-1)/programmed death ligand 1 (PD-L1) axis has produced clinically meaningful activity in unresectable HCC and is being explored in the neoadjuvant setting (13). Nevertheless, across multiple pivotal studies, immune-checkpoint inhibitor (ICI) monotherapy yielded objective response rates of only 15%–20% (14, 15). Greater response and survival metrics are generally observed with combination treatments. For example, in the IMbrave150 phase 3 trial, adding targeted therapy to immunotherapy extended median overall survival from 13.4 to 19.2 months in unresectable, advanced HCC (16). By contrast, IMbrave050 reported that, among patients treated postoperatively, recurrence during follow-up remained near 50% despite combined immunotherapy and targeted therapy (17). Furthermore, several countries participating in the trial continue to experience rising mortality from cirrhosis-associated HCC (4). An important contributing factor is the presence of chronic liver disease, particularly cirrhosis, accompanying HCC and remodeling the hepatic immune environment, thereby promoting tumor growth and recurrence. The cirrhotic milieu fosters immune suppression, providing “fertile soil” for immune escape, with cirrhosis-conditioned hepatocytes or progenitor cells serving as “susceptible seeds” for malignant transformation. Consequently, elucidating how cirrhosis alters immune regulation, along with developing therapies that address both the tumor and the cirrhosis-shaped immune milieu, could lead to improved treatment efficacy. As fibrosis advances, the diversity and activity of immune cells undergo profound changes, forming a microenvironment that fosters fibrogenesis while dampening antitumor immune responses. This review synthesizes current evidence on how cirrhosis alters hepatic immune populations and influences immunotherapy responses, while highlighting candidate targets and innovative combination strategies tailored to HCC arising in the cirrhotic liver.

## 2 Cirrhosis-driven remodeling of the immune microenvironment and its impact on HCC immunotherapy

Against a backdrop of cirrhosis, the hepatic immune system undergoes remodeling, with key immune-cell populations including dendritic cells (DCs), T cells, macrophages, and neutrophils. Alterations in the abundance, spatial distribution, and functions of these cell populations interact, collectively shaping the sensitivity and clinical response of HCC to immunotherapy (**Table 1**).

**Table 1:** Changes in key immune cells associated with liver diseases including cirrhosis and hepatocellular carcinoma.

Cell type	Model	Related changes	Key molecules/Signaling pathways	References
Dendritic Cells	Peripheral blood of patients with acute decompensated cirrhosis (AD) and stable cirrhosis (SC)	Decreased in DC count. Decreased in cDC count, increased in pDC percentage	IL-6, IL-10↑	(18)
	Peripheral blood of patients with acute-on-chronic liver failure	Reduced antigen-presenting capacity of MoDCs	HLA-DR, CD86, CD54, IFN-γ↓	(19)
	Mice hepatocellular carcinoma cell line Hepa	TGF-β upregulates PD-L1 expression in dendritic cells via the STAT3 pathway and suppresses T cell antitumor immunity	TGF-β, STAT3, PD-L1	(20)
	HCC tissues of patients and mice HCC models	eADO-stimulated pDCs promoted the increase of IL-10, IDO), and PD-L1, the expansion of regulatory T cells and the exhaustion of CD8^+^T cell	eADO, IL-10, IDO, PD-L1	(21)
	Dendritic cells isolated from human liver tissue	Upon TLR4 ligation, liver dendritic cells secrete IL-10, promoting T cell hyporesponsiveness and facilitating the generation of suppressive Treg and Th2 cells	IL-10	(22)
	Rats dendritic cells co-transfected with IL-10 and TGF-beta 1 genes *in vitro*	Co-transfection of IL-10 and TGF-β1 in immature dendritic cells led to downregulation of MHC II, CD80, and CD86 expression and induced T cell hyporesponsiveness	IL-10, TGF-β1	(23)
T cells	CD8^+^T cells from peripheral blood, ascites and liver explants from patients with liver cirrhosis	Inhibited cytotoxic effects and induced phenotypic and functional abnormalities in monocytes and neutrophils	PD-1,CTLA-4,TIM-3, LAG-3↑	(24)
	CCL4-induced liver fibrosis mice	Increased CD38^+^HLA-DR^+^CD8^+^T cells, and exacerbated liver injury	JAK/STAT5 PI3K/mTOR	(25)
	*In vitro* co-culture of primary mouse HSCs and T cells	Inhibited the proliferation of CD4^+^ and CD8^+^ T cells and the production of IFN-γ	TGF-β1	(26)
	Patients with acute decompensated liver cirrhosis	Mitochondrial dysfunction, enhanced glycolysis, and pentose phosphate pathway	Long-chain acylcarnitine palmitoylcarnitine↑ CXCL8, IL-8↑	(27)
	Cohort of patients with cirrhosis stratified by stage	CD4+, CD8+ T cell dysfunction worsens with the progression of liver cirrhosis	None	(28)
	Dataset containing gene expression profiles of patients with liver cirrhosis and healthy controls	CD8+ T cell subsets exhibited heterogeneity in liver cirrhosis, exhausted T (Tex) cells were increased in cirrhosis, and CXCL13+ Tex cells display an exhausted phenotype associated with immune dysregulation and advanced disease.	CXCL13	(29)
	Peripheral blood and ascites fluid from patients with decompensated cirrhosis.	Identified CXCR6^+^CD69^+^ CD8^+^ T cells and exhibit a chronically activated bystander phenotype with innate-like functions. They are associated with disease severity	CXCR6^+^CD69^+^	(30)
Macrophage	Tlr4-chimeric mice	KCs were activated and induced a pro-inflammatory response	LPS-TLR4-NF-κB	(31)
	Hepatic IRI (Ischemia-Reperfusion Injury) mice	Elevated HMGB1 promoted inflammatory responses in the liver by activating macrophages and other cells, leading to hepatocyte damage	ASC/caspase-1/IL-1β	(32)
	HCC tissues of patients	Increased fatty acid oxidation induced M2 polarization of macrophages	receptor-interacting protein kinase 3 (RIPK3)↓	(33)
	Blood from patients with liver cirrhosis and CCL4-induced liver fibrosis mice	A TREM2^+^CD9^+^ scar-associated macrophage subpopulation existed in human liver cirrhosis tissue	TREM2	(34)
	CCL4-induced liver fibrosis mice	KCs secreted TGF-β, driving HSC activation and promoting fibrosis.	CXCL6, TGF-β↑	(35)
	HCC tissues of patients	The expression of B7-H1 (PD-L1) on KCs was elevated, and PD-1^+^ CD8^+^ T cells were enriched in the tumor area and functionally inhibited	B7-H1(PD-L1)	(36)
Neutrophil	STAM model of NASH mice	Early neutrophil infiltration and NET formation, followed by macrophage influx, production of inflammatory cytokines, and progression to HCC	NET	(37)
	CCL4-induced liver injury and fibrosis mice	PAD4 drives NET formation in acute liver injury but may be unrelated to NET formation in chronic liver injury	PAD4, NET	(38)
	MASH diet mice with alcohol binges	Nod-like receptor protein 3 (NLRP3) could detect increased NETs, inducing HSCs and pro-inflammatory monocytes to produce a pro-fibrotic phenotype	NLRP3	(39)
	HCC tissues of patients and mice HCC models	The CLCF1/CXCL6/TGF-β axis upregulates N2-type neutrophil recruitment in HCC and correlates with poor prognosis	CLCF1/CXCL6/TGF-β	(40)
	HCC tissues of patients and mice HCC models	PLAUR^+^ neutrophils influence CD8^+^ T cell and macrophage function to form an immunosuppressive microenvironment	PLAUR	(41)

The symbol “↓” means “decrease”. The symbol “↑” means “increase”.

### 2.1 DCs

DCs act as the central link between innate defenses and adaptive responses. By capturing, processing, and presenting antigens, they prime naïve T cells and launch antitumor immunity. When DCs are dysfunctional, antigen display and immune priming decline, weakening clinical responses to immunotherapy (42).

Cirrhosis, a condition in which chronic inflammation, immune activation, and immune suppression coexist, markedly reshapes DC biology. Multiple clinical investigations describe lower peripheral DC counts together with altered subset balance. For example, Cardoso and colleagues reported that individuals with acute decompensated (AD) or stable cirrhosis (SC) had fewer total DCs, a higher plasmacytoid/classical DC (pDC/cDC) ratio, and elevated concentrations of interleukin (IL)-6 and IL-10 compared with healthy controls—findings consistent with simultaneous inflammation and suppression (18). In acute-on-chronic liver failure (ACLF), functional impairment is even greater. Wu et al. showed that monocyte-derived DCs (MoDCs) downregulate human leukocyte antigen (HLA)-DR, CD86, and CD54, display reduced antigen-presenting and T-cell-stimulating capacity, and produce inadequate levels of interferon (IFN)-γ (19). With advancing cirrhosis, maturation and priming functions become increasingly restricted. Notably, cultured MoDCs obtained from patients can reacquire mature phenotypes, characterized by expression of CD80, CD86, and HLA-DR, and effectively activate tumor antigen-specific T cells, indicating that their intrinsic antigen-presenting capacity is preserved (43).

Within the HCC microenvironment, cDCs are depleted and pDCs are functionally compromised. Upregulation of IL-10 and PD-L1 promotes tolerogenic DC states that dampen CD8^+^ T-cell activation and antitumor activity (20, 21, 44). The cirrhotic liver milieu, enriched in IL-10 and transforming growth factor (TGF)-β, further suppresses DC maturation and antigen presentation while expanding regulatory T cells (Tregs), thereby limiting effector responses (45). TGF-β can also drive immature DCs to generate antigen-specific CD8^+^ Tregs that inhibit other effector T cells and foster tumor tolerance (46).

However, research indicates that in CCL4-induced mouse models of liver fibrosis, fibrotic liver dendritic cells (FLDCs) exhibit a distinct and mature phenotype compared to normal liver dendritic cells (NLDCs). Expression of MHC II and CD40, the key molecules for antigen presentation, is upregulated in FLDCs. FLDCs exhibit significantly enhanced immunogenicity in both *in vivo* and *in vitro* experiments, which correlates with their secretion of TNF-α. FLDCs more effectively activate NK cells, inducing higher levels of IFN-γ production and increased cytotoxicity, while also enhancing the proliferation and activation of CD4^+^ and CD8^+^ T cells. Immunization with FLDCs completely prevents tumor development in mice (47). Jiao et al. demonstrated that following CCL4-induced liver fibrosis in mice, the number of hepatic cDCs and pDCs significantly increased during the early fibrotic regression phase after drug withdrawal, playing a crucial role in fibrosis reversal. Depletion of DCs in CD11c-DTR mice markedly delayed fibrosis regression and reduced clearance of activated hepatic stellate cells. Conversely, both Fms-like tyrosine kinase-3 ligand (Flt3L) expanded DCs and purified DCs accelerated fibrosis regression upon adoptive transfer. This effect primarily involved DC-secreted MMP-9 promoting extracellular matrix degradation (48).

Taken together, cirrhosis reduces DC abundance, constrains maturation under immunoregulatory cues, and promotes tolerogenic phenotypes, establishing a suppressive immune niche. These changes blunt responses to immunotherapy but also reveal therapeutic opportunities to restore DC function or relieve inhibitory pathways such as PD-L1 and TGF-β, with the goal of improving clinical benefit.

### 2.2 T cells

#### 2.2.1 Effects of cirrhosis on T-cell function

Persistent inflammatory signals and metabolic imbalance in cirrhosis extensively reprogram T-lymphocyte phenotypes and functions. Continuous antigenic stimulation, together with distortion of the sinusoidal structure and engagement of hepatic tolerance pathways, pushes CD8^+^ and CD4^+^ T-cell compartments toward chronic activation and eventual exhaustion. Peripheral blood from individuals with cirrhosis has been shown to exhibit elevations in PD-1, cytotoxic T-lymphocyte-associated protein 4 (CTLA-4), and T-cell immunoglobulin and mucin domain-containing 3 (TIM-3) on CD8^+^ cells, with parallel loss of cytotoxic capacity and reduced IFN-γ secretion (24), indicating attenuated T-cell activity. Niehaus and colleagues further observed that dysfunction intensifies as cirrhosis advances (28). TGF-β can directly limit proliferation and IFN-γ production in both CD8^+^ and CD4^+^ subsets, thereby curbing activation and antitumor immunity (26).

Single-cell profiling demonstrates substantial heterogeneity within the CD8^+^ T-cell pool in cirrhosis, with pronounced enrichment of coexisting effector memory (Tem) and exhausted (Tex) T-cell populations alongside upregulated PD-1 expression (29). IL-15 can provoke bystander activation of CD38^+^HLA^−^DR^+^CD8^+^ T cells and aggravate hepatic injury through the Janus kinase (JAK)/signal transducer and activator of transcription (STAT)5 and phosphoinositide 3-kinase (PI3K)/mechanistic target of rapamycin (mTOR) pathways (25). Recently reported single-cell and bulk transcriptomic datasets show clonal expansion of intrahepatic and circulating CD8^+^ cells, contraction of T-cell receptor repertoire diversity, and reinforced exhaustion programs marked by thymocyte selection-associated high mobility group box (TOX), T-cell immunoreceptor with Ig and ITIM domains (TIGIT), and PD-1, all consistent with impaired immunity (49).

Metabolic conditions are also profoundly altered in cirrhosis. Heightened competition for glucose and amino acids, together with disordered handling of lactate and fatty acids, exposes circulating and hepatic T cells to nutrient scarcity and metabolic stress. In liver tissue samples from cirrhotic patients, IDO protein expression is upregulated and accompanied by increased serum tryptophan metabolism to kynurenine (Kyn). The kynurenine pathway (KP) is activated and closely correlates with the severity of cirrhosis (50, 51). Furthermore, in a mouse model of hepatocellular carcinoma, Kyn was shown to induce PD-1 upregulation in CD8^+^ T cells by activating the aryl hydrocarbon receptor (AhR), thereby evading immune killing and reducing the efficacy of immunotherapy (52). Concurrently, peripheral MDSC expansion in cirrhotic patients increases arginase release while decreasing L-arginine concentration (53). This restricted amino acid supply downregulates T-cell CD3ζ expression and suppresses T-cell proliferation and immune function (54). Furthermore, reduced glucose uptake and weakened activation of the AKT/mTOR pathway during cirrhosis impair the proliferation and differentiation capacity of T cell-dependent activated B cells, limiting immune function (55). Downregulated glycolysis may impair certain CD8^+^ T cell functions via the mTOR pathway and adversely affect IFN-γ production (56). In a mouse sarcoma model, tumor glucose consumption was shown to metabolically constrain T cells, diminishing their mTOR activity, glycolytic capacity, and IFN-γ production. This metabolic suppression impairs T cell function and promotes exhaustion, thereby accelerating tumor progression (57). As disease progresses from steatosis to fibrosis and ultimately to HCC, CD8^+^ T-cell bioenergetics decline substantially, with a reduction in oxidative phosphorylation (OXPHOS) and in glycolytic flux (58). Through analyzing the mitochondrial function of white blood cells in patients with cirrhosis, a study found that the number of mitochondria within white blood cells increases but their volume decreases, and the gene expression of glycolysis and pentose phosphate pathway is upregulated (27). These changes directly indicate that immune cells, including T cells, undergo functional metabolic damage in the late stage of cirrhosis, inhibiting their immune function.

Enrichment of CXCR6^+^CD69^+^CD8^+^ T cells has also been identified in ascitic fluid from patients with cirrhosis. These cells display features that have been implicated in the development of nonalcoholic steatohepatitis-associated HCC, namely a highly activated bystander phenotype and intrinsic tissue-damaging properties (30, 59).

#### 2.2.2 Adverse implications for HCC immunotherapy

Cytotoxic T lymphocytes are principal executors of antitumor immunity in HCC, eliminating transformed targets through antigen recognition and direct killing (60, 61). They also secrete mediators such as tumor necrosis factor (TNF)-α, IFN-γ, and granzyme B (GzmB), which promote tumor cell death (62). In patients with cirrhosis-associated HCC, the immune context shaped by cirrhosis creates several barriers to effective T-cell-based immunotherapy. First, Tex cells display high levels of PD-1 and TIM-3 yet recover only modest function. In one study of cirrhosis, CD8^+^ T cells showed increased expression of TIM-3 and lymphocyte activation gene 3 (LAG-3), with a larger fraction coexpressing PD-1 and TIM-3 (63). These observations suggest that, even under alleviation of PD-1 signaling, metabolic stress, mitochondrial injury, and fixed exhaustion programs can still limit reinvigoration.

T-cell bioenergetics are tightly connected to fibrosis in HCC. Within tumors, lymphocytes and cancer cells compete for lipids, impairing T-cell performance and facilitating malignant progression (64). Concurrently, both compartments vie for glucose, leading to scarcity and accumulation of lactate, which suppresses T-cell activity and promotes regulatory programs driven by Tregs (65). Cancer cells further weaken immunity by consuming essential amino acids and producing harmful metabolites. For instance, high expression of solute carrier family 7 member 11 (SLC7A11) enables preferential cystine uptake by tumor cells, promoting T-cell exhaustion and ferroptotic vulnerability, reducing memory formation and cytokine secretion, increasing PD-1 and TIM-3 expression, and heightening oxidative stress (66). Targeting these metabolic circuits may therefore increase sensitivity to immunotherapeutic and cytotoxic drugs.

Finally, as described by Niehaus et al., enrichment of bystander type CXCR6^+^CD8^+^ cells in ascitic fluid represents a population that consumes resources without participating in antigen-specific tumor control, further diminishing the effectiveness of ICIs (30). Overall, exhaustion, metabolic repression, and Treg expansion in cirrhosis coordinate to restrict the capacity of ICIs to reactivate T cells, revealing actionable targets to improve outcomes in cirrhosis-associated HCC.

### 2.3 Macrophages

#### 2.3.1 Effects of cirrhosis on macrophage function

Cirrhosis, representing an advanced consequence of long-standing liver injury with recurrent inflammation, cell death, and progressive scarring, profoundly remodels the hepatic innate immune milieu and strongly impacts macrophage compartments, notably Kupffer cells (KCs) and monocyte-derived macrophages (MoMφs). In early disease, disruption of the intestinal barrier allows gut-derived lipopolysaccharide to reach the liver through the portal circulation, where it engages KCs via the Toll-like receptor (TLR)4–nuclear factor (NF)-κB pathway and elicits proinflammatory signaling (31). Concurrently, damage-associated molecular patterns (DAMPs) released from necrotic hepatocytes, such as high mobility group box 1 (HMGB1) are sensed by KCs, which then secrete IL-1β, TNF-α, C-C motif chemokine ligand (CCL)2, and C-X-C motif chemokine ligand (CXCL)8 and activate the inflammasome through TLR4, thereby amplifying tissue inflammation and worsening hepatic injury (32, 67).

Concurrently, Ly6C^hi^ MoMφs display proinflammatory profiles characterized by elevated levels of mediators such as TNF-α, IL-1β, IL-6, CCL2, and CCL5, together with profibrogenic signals including IL-13. Continued stimulation through chemokine receptor (CCR)2/CCR5 in cirrhotic tissue promotes their polarization toward M2 or scar-associated macrophage states, which suppress T-cell function, remodel the extracellular matrix, and directly activate hepatic stellate cells in a TGF-β-dependent fashion, thereby driving the shift from inflammation to fibrosis (68–70). In contrast, Ly6C^lo^ MoMφs exert antifibrotic effects (71). In experimental models using repeated CCl4 exposure or a methionine- and choline-deficient diet (MCD), inhibition of CCL2 limits the influx of Ly6C^hi^ cells, increases the proportion of Ly6C^lo^ cells, and accelerates fibrosis regression (68). With persistent injury, loss of M1-like macrophages and other leukocytes favors expansion of M2-like populations that, under chronic cytotoxic stress, release IL-4, IL-10, and TGF-β as protective mediators (72). Polarized KCs increase expression of CD163, CD206, and Arg-1 while secreting IL-10, TGF-β, and vascular endothelial growth factor (VEGF), collectively diminishing antigen presentation and promoting immune tolerance and angiogenesis (73, 74). In parallel, the TGF-β/Smad pathway heightens hepatic stellate cell activation and collagen synthesis, resulting in matrix remodeling and increased tissue stiffness (75). In advanced cirrhosis, functional roles diverge, with KCs showing a tendency to maintain immune homeostasis and tolerance while MoMφs contribute to matrix turnover and scar formation (76, 77). Single-cell studies in human cirrhotic liver have identified a triggering receptor expressed on myeloid cells 2 (TREM2)^+^CD9^+^ scar-associated macrophage population, largely derived from circulating monocytes, that expresses abundant TGF-β and promotes collagen deposition and extracellular matrix accumulation (34).

Hypoxia arising from altered sinusoidal hemodynamics further reprograms macrophage metabolism. The mTOR complex 2 (mTORC2)–interferon regulatory factor 4 (IRF4) axis and peroxisome proliferator-activated receptor (PPAR)-related pathways are key drivers of M2 activation coupled to fatty acid oxidation (FAO) and OXPHOS (78, 79). Consistently, tumor-associated macrophages (TAMs) within cancers often rely on FAO, which supports M2 polarization and strengthens immunosuppressive activity (33).

At the signaling level, the TLR4–NF-κB–STAT3 cascade is central to KC reconfiguration in cirrhosis. Chronic exposure to pathogen or damage-associated cues sustains TLR4 activity, while proinflammatory cytokine production coexists with STAT3-mediated feedback, gradually transitioning early inflammatory responses toward later immune suppression (80, 81). In addition, KC-derived TGF-β and PD-L1 respectively promote persistent activation of hepatic stellate cells and dysfunction of CD8^+^ T cells, creating a pathologic niche where tolerance and fibrosis are reinforced (35, 36). In summary, signals originating from the gut, necrotic tissue, and chronic hypoxia drive KCs from an M1 proinflammatory program toward an M2 immunoregulatory state, effecting a shift from amplification of inflammation to maintenance of immune tolerance.

#### 2.3.2 Adverse implications for HCC immunotherapy

During cirrhosis, hepatic macrophages encompassing resident KCs and MoMφs shift from proinflammatory programs in early stages to immunoregulatory and tissue-reparative states as the disease progresses. This transition provides much of the biological basis for the reduced effectiveness of immunotherapy once HCC emerges.

First, antigen presentation and the display of costimulatory ligands by macrophages are curtailed in the cirrhotic liver. Single-cell analyses of fibrotic and cirrhotic specimens have identified TREM2^+^CD9^+^ scar-associated macrophages that originate largely from circulating monocytes. These cells are suppressive, weaken effective antigen display, and limit activation of T lymphocytes, thereby facilitating tumor progression (34, 82). Because initial priming of CD8^+^ and CD4^+^ effectors is inadequate, treatment with PD-1 or PD-L1 inhibitors often only partly restores T-cell function.

Second, cirrhosis skews macrophages toward high output of immunosuppressive cytokines, include IL-10 and TGF-β, and increased PD-L1 expression, establishing an immune “OFF” configuration. Reviews indicate that TAMs in HCC and other solid cancers are predominantly M2-like, characterized by secretion of IL-10 and TGF-β and expression of PD-L1 and CSF1R, which collectively restrain CD8^+^ T-cell proliferation, reduce IFN-γ production, and promote T-cell exhaustion. Macrophage PD-L1 directly mediates checkpoint-based inhibition of T cells (83–85). In cirrhosis, chronic stimulation by pathogen-associated and damage-associated signals sustains the TLR4–NF-κB pathway, and STAT3-mediated feedback progressively biases these cells toward tolerance, leaving T cells less responsive to subsequent checkpoint blockade (86, 87).

Third, macrophages drive remodeling of the extracellular matrix and abnormal angiogenesis, impeding the entry of effector lymphocytes. Under these conditions, even with PD-1 or PD-L1 blockade, T cells struggle to traverse fibrotic tissue and remain sparsely distributed within tumors, limiting their activity (88, 89).

Fourth, metabolic rewiring toward FAO and OXPHOS in macrophages enhances IL-10 and TGF-β production and augments PD-L1 expression. This program suppresses the proliferative and cytotoxic capacities of tumor-infiltrating CD8^+^ T cells and diminishes the benefit of ICI therapy (33). Enzymes such as arginase 1 and indoleamine dioxygenase deplete essential amino acids and promote lactate accumulation, producing metabolic paralysis and functional exhaustion in T cells (90–92). Macrophage-derived mediators including IL-10, TGF-β, and indoleamine-2,3-dioxygenase 1 (IDO1) also recruit myeloid-derived suppressor cells (MDSCs) and Tregs, reinforcing a coupled metabolic and immune-inhibitory circuit (93, 94).

Evidence also points to a countervailing role for macrophages. When KCs are activated, they release IL-12, which stimulates liver-resident NK cells and NK T lymphocytes to produce the cytokine IFN-γ, thereby licensing hepatic T cells to join the antitumor defense (95, 96). Thus, even within cirrhosis, macrophages are not confined to tolerance and suppression of T-cell activity.

Taken together, these mechanisms of impaired antigen presentation, intensified cytokine and checkpoint signaling, strengthened structural barriers, and deepened metabolic suppression converge to form a macrophage-centered suppressive network that lowers both the response rate and durability of immunotherapy in HCC.

### 2.4 Neutrophils

#### 2.4.1 Effects of cirrhosis on neutrophil function

In cirrhosis, iterative cycles of injury and repair, rising portal pressure, and matrix scarring, alongside persistent elevations in mediators such as IL-8 and granulocyte colony-stimulating factor (G-CSF), and regulatory signals like TGF-β, collectively reprogram neutrophils in both blood and liver, altering their phenotype and function. Patients show broad alterations across phagocytosis, bactericidal capacity, chemotaxis, degranulation, production of reactive oxygen species (ROS), and formation of neutrophil extracellular traps (NETs) (97, 98). Translational studies of cirrhosis report higher circulating levels of trap markers, including histone H3 citrullinated-DNA (H3Cit-DNA) and myeloperoxidase (MPO)-DNA complexes (37), alongside reduced phagocytic and killing abilities (99), disturbed control of oxidative bursts (100), increased tissue infiltration with poor effector killing, and abnormal apoptosis (101).

At a mechanistic level, NET generation depends on a program of peptidylarginine deiminase 4 (PAD4)-driven histone citrullination and release of granule enzymes, which is upregulated in fibrosis and cirrhosis (38, 102). These traps can directly activate the AIM2 inflammasome in macrophages, driving IL-1β-dependent inflammation and pyroptosis, which then enhances fibrogenic activity in fibroblasts and hepatic stellate cells (39, 103). In parallel, neutrophil elastase (NE), matrix metalloproteinases (MMP), and adhesion receptors remodel the extracellular matrix and increase stromal density (104). Prolonged inflammation with sustained G-CSF signaling biases the lineage toward low-density immunosuppressive populations that release abundant ROS and inhibitory cytokines, thereby diminishing antigen display and cytotoxicity and creating conditions favorable for tumor immune escape (105, 106).

#### 2.4.2 Adverse implications for HCC immunotherapy

These neutrophil-driven changes lower the effectiveness of ICI therapy in HCC through several routes. Excessive NET formation builds physical obstacles within the tumor stroma (107) that hinder immune cell entry (108), while activation of cancer-associated fibroblasts (CAFs) (109) and stimulation of the matrix metalloproteinase and VEGF pathways (110, 111) further restrict drug penetration and blunt effector T-cell activity. Specific tumor-associated neutrophil (TAN) subsets, such as plasminogen activator urokinase receptor (PLAUR)^+^ and CD10^+^ cells, can express PD-L1 and release high levels of ROS and suppressive cytokines, pushing CD8^+^ T cells toward exhaustion and functional decline and thereby lowering responses to anti PD-1 and anti PD-L1 therapy (41, 112, 113). In cirrhotic HCC, heterogeneity and metabolic shifts favor a pro-tumor N2-like state (114). Higher CXCL9 levels correlate with N1 polarization and improved responses to checkpoint blockade (115, 116), implying that low expression may limit this program, while CXCL9 augmentation could drive N1 conversion, enhance T-cell function, and bolster outcomes.

In summary, neutrophil dysfunction in cirrhosis appears to reduce immunotherapy success through direct immune suppression and indirect remodeling of the matrix and tissue milieu. Strategies that raise therapeutic efficacy by dismantling traps with deoxyribonuclease (DNase), inhibiting resistance-linked neutrophil markers such as PLAUR, or promoting N1 polarization warrant consideration.

## 3 Targets and strategies to improve immunotherapeutic efficacy in HCC

### 3.1 DCs

As previously mentioned, the cirrhosis and HCC milieu is commonly associated with fewer DCs, delayed or incomplete maturation of antigen-presenting subsets, and diminished expression of costimulatory ligands. Together, these changes weaken antigen display and the priming of naive T lymphocytes, implying that releasing microenvironmental brakes could be an effective first step toward rescuing DC activity. Robust antitumor immunity in HCC depends strongly on the cDC1 lineage, noted for cross presentation and licensing CD8^+^ T cells; therefore, preferential restoration or supplementation of cDC1 function is a central avenue to strengthen immunity (117). Furthermore, experimental work shows that inhibitory cues within the tumor bed, including VEGF and IL-10, directly disrupt DC maturation and differentiation. Neutralization of VEGF with VEGF-Trap treatment improves DC development, supporting a strategy that lifts suppression to recover function (118, 119). Given the mechanisms that drive PD-L1 expression on DCs in HCC patients, blocking PD-L1 on TAMs could relieve direct inhibition of effector T cells while reducing negative signaling on DCs themselves, thereby enhancing antigen presentation and co-stimulation. Such a maneuver could serve as a valuable element of combination therapy (120, 121). In parallel, amplifying costimulatory pathways with a bispecific antibody such as DuoBody CD40×4-1BB could offset downregulation of CD86, increase DC-mediated T-cell activation, and expand effector populations, demonstrating another feasible route to activation (122).

Improving the recruitment and spatial distribution of intratumoral DCs is also important. When paired with PD-1 blockade, inhibition of CXCR4-dependent chemotactic defects increases entry of DCs and effector lymphocytes into tumor tissue and augments responses (123). From the perspective of innate sensing, activating the cyclic GMP-AMP synthase (cGAS)-stimulator of interferon genes (STING) pathway directly promotes DC maturation and induces type I IFN programs, offering a potent *in vivo* trigger of function (124). Iron–manganese (Fe–Mn) bimetal nanovaccines can provoke pyroptosis, releasing damage-associated signals and dsDNA that activate the cGAS-STING pathway, with further potentiation by Mn. This cascade drives type I IFN and inflammatory cytokine production, promotes DC maturation, enhances antigen presentation, and recruits large numbers of CD8^+^ T cells to the tumor, thereby intensifying antitumor responses (125). Agonists of TLR4 and TLR7/8 also stimulate DC maturation and are widely used as vaccine adjuvants or within nanodelivery systems to raise the efficiency of antigen presentation and T-cell priming (126). Advances in nanotechnology now enable co-encapsulation of antigens with STING or TLR agonists and targeted delivery to the tumor bed or draining lymph nodes, focusing on *in vivo* DC activation and improving the quality of priming (127).

Ex vivo-generated DC vaccines, loaded with tumor antigens and reinfused back into patients, can provide a direct supply of functional antigen-presenting cells. When these vaccines are combined with radiotherapy or necrosis-inducing local procedures such as TACE, the therapy-induced release of tumor antigens can be exploited to amplify systemic immunity. Several clinical trials have already been completed (128, 129), including one in which adding a DC vaccine to cyclophosphamide conditioning and TACE enhanced antigen-specific responses and significantly lengthened survival. Personalized approaches using neoantigens have also shown the capacity to elicit specific CD8^+^ T-cell responses (130). Concurrently, DC vaccines used alone in HCC have shown generally good safety and measurable immunogenicity but modest objective responses, suggesting that future benefit will most likely come from combinations with ICIs, *in vivo* DC-activating adjuvants, or local therapies (131).

An especially promising strategy involves integrating individualized neoantigen vaccines with *in vivo* DC activators such as STING/TLR agonists and immune checkpoint blockade. This tripartite design simultaneously addresses the three major limitations of antigen supply, DC activation, and ongoing immune suppression, and may deliver more powerful regimens for patients with cirrhosis-associated HCC (132).

### 3.2 T cells

In cirrhosis, strengthening T-cell activity for HCC therapy is best pursued with a layered, multitarget combination strategy. Key objectives include counteracting exhaustion programs, restoring mitochondrial and overall metabolic fitness, dampening suppressive cells and mediators, and refining local delivery to minimize liver toxicity.

Because Tex cells frequently coexpress several immune checkpoints, simultaneous blockade at two or more nodes, such as PD-1 together with TIM-3/LAG-3, liberates CD8^+^ T cells more effectively than PD-1 inhibition alone, with synergistic signals observed in both preclinical systems and early clinical studies (133, 134). However, it is rare for removal of inhibitory receptors alone to fully rescue function. Tex cells also exhibit damaged mitochondria and rewired metabolism. Enforced expression of proliferator-activated receptor gamma coactivator 1-alpha (PGC-1α) increases mitochondrial biogenesis, improves the fitness of CD8^+^ populations, and heightens antitumor efficacy—observations supported in both chimeric antigen receptor T-cell (CAR-T) and tumor-infiltrating lymphocyte models (135, 136). As cytokine support, short courses or locoregional dosing of IL-15, including engineered hyper-IL-15, can expand CD8^+^ effectors and boost their function, with antitumor activity reported in liver metastasis and spontaneous HCC models, suggesting compatibility with ICI therapy or cell-based treatments (137, 138).

Cirrhosis also brings expansion of Tregs and other suppressive myeloid populations, including MDSCs and immunoregulatory TAMs. Selectively depleting or disabling Tregs with low-dose cyclophosphamide can transiently lift the brakes on effector cells, strengthening systemic antitumor responses (139). In parallel, interrupting the myeloid suppressive network—either by reprogramming macrophages through inhibition of colony-stimulating factor 1 (CSF1) or its receptor (CSF1R), or by blocking the recruitment and function of MDSCs—can indirectly enhance T-cell infiltration and effector potency (140).

TGF-β sits at the intersection of fibrosis and immune suppression, making it an attractive target. Small-molecule inhibitors of TGF-β receptor I, such as galunisertib, have shown antitumor signals in early HCC trials and, in animal models, reduce fibrosis-related stromal barriers while improving immune cell infiltration. When combined with sorafenib, this approach has yielded acceptable safety and longer overall survival (141), supporting its inclusion within combinatorial regimens.

Epigenetic modulators, including histone deacetylase (HDAC) inhibitors, can raise tumor immunogenicity, enhance antigen presentation, and synergize with checkpoint blockade. With careful attention to dosing and route, this avenue may further improve outcomes (142). Finally, local and regional immune activation deserves more emphasis. Intratumoral oncolytic viruses as single agents have demonstrated antitumor activity with low systemic toxicity. In HCC, related studies indicate that pairing such local therapy with systemic ICIs can amplify whole-body antitumor immunity (143), offering a practical path to lift the effectiveness of immunotherapy in patients with cirrhosis.

### 3.3 Macrophages

Macrophage reprogramming in cirrhotic livers—a shift that favors tolerance and supports HCC—has made these cells attractive therapeutic targets for boosting responses to immunotherapy. Concurrent inhibition of CCR2 and CCR5 with agents such as cenicriviroc limits the influx of suppressive macrophages and promotes fibrotic regression in hepatic models (144, 145). Signaling through CSF1 and CSF1R maintains M2-skewed phenotypes; blocking CSF1R can lessen TAM-driven immunosuppression and act synergistically with PD-1 blockade (146). Cirrhosis-enriched TREM2^+^CD9^+^ scar-associated macrophages, largely originating from circulating monocytes, contribute to matrix remodeling and immune suppression; anti-TREM2 therapy paired with ICIs restored cytotoxic T-cell activity in preclinical studies (147). The interaction between CD47 on tumor cells and signal regulatory protein alpha (SIRPα) on phagocytes delivers an antiphagocytic signal. Consistently, interrupting this pathway via antibodies against CD47 or SIRPα enhances macrophage engulfment, improves cross-presentation, and augments PD-1 therapy (148, 149). In HCC models, the anti-CD47 antibody B6H12 stimulates macrophage-mediated clearance, restrains tumor growth, and increases chemotherapy efficacy (150). Another innate checkpoint, the CD24–Siglec-10 axis, suppresses myeloid activation, with its blockade lifting myeloid quiescence and strengthening antitumor immunity (151). Kinases of the TAM family, MERTK and AXL, help preserve immunosuppressive TAM programs; inhibitors of these receptors combined with checkpoint blockade show signs of enhanced immune activity (152, 153). Myeloid PI3Kγ functions as a hub for suppressive transcriptional circuits. Inhibition of PI3Kγ with eganelisib repolarizes TAMs and MDSCs toward inflammatory states, restores T-cell function, and can overcome resistance when added to ICI therapy (154). Because persistent TLR4 signaling sustains tolerogenic macrophage states in cirrhosis, TLR4 inhibition reduces immunosuppressive mediators and improves antitumor responses, making it a plausible partner in combination regimens (155).

Macrophage metabolism is also actionable. Restraining lactate production or export—through targets such as lactate dehydrogenase A (LDH-A) or monocarboxylate transporter (MCT)4—can correct suppressive macrophage bioenergetics and heighten the effectiveness of ICI therapy (156). Both hypoxia and pathologic angiogenesis hinder T-cell trafficking and maintain suppressive myeloid cell pools, and pairing anti-VEGF antibodies or angiopoietin-2 (Ang2) blockade with ICIs promotes vascular normalization alongside immune activation. Notably, atezolizumab combined with bevacizumab improved overall and progression-free survival in HCC (157, 158).

### 3.4 Neutrophils

As cirrhosis evolves into HCC, persistent inflammation, stromal remodeling, and a locally suppressive immune context within both the tumor niche and surrounding liver tissue reshape neutrophil behavior and polarization, ultimately diminishing T cell-driven antitumor activity. These observations provide a strong rationale for therapeutically modulating neutrophils to improve the performance of immunotherapy, a concept supported by preclinical data.

The generation of NETs represents a key pathogenic output of this lineage. In HCC, such traps facilitate immune escape, in part by fostering Treg infiltration (159). Pharmacologic dismantling with DNase I and inhibition of PAD4 using agents such as GSK484 reduce NET burden and enhance T-cell entry, yielding greater antitumor effects when paired with PD-1 or PD-L1 blockade (37, 160). Consequently, interfering with NET formation or persistence represents an important intervention point against neutrophil-mediated immune evasion (161, 162).

Neutrophil trafficking can also be targeted. In cirrhosis and HCC, chemokines including IL-8 and CXCL family members, together with their receptors CXCR1 and CXCR2, orchestrate marrow egress, circulation, and tumor homing. Inhibition of CXCR2 with AZD5069 combined with ICI therapy expands antitumor neutrophil populations and lowers tumor burden in experimental HCC (163). Thus, blocking the IL-8–CXCR1/CXCR2 axis represents a potent means to increase immunotherapy sensitivity. Resistance to ICI therapy is further linked to the accumulation of low-density or otherwise suppressive neutrophils, such as CD10^+^ALPL^+^ and PLAUR^+^ subsets, which produce ROS, arginase 1, and PD-L1, driving CD8^+^ T-cell exhaustion (112). PLAUR^+^ neutrophils are enriched in nonresponders to PD-1 therapy and predict poor outcomes (41). Selective depletion or functional neutralization of these subsets by targeting markers such as PLAUR or CD10/ALPL may reverse suppression and improve therapeutic efficacy. In parallel, neutrophil-expressed checkpoints and their metabolic wiring remain actionable. Lactate-rich, acidic tumor conditions rewire neutrophils to induce expression of PD-L1, cyclooxygenase 2 (COX-2), and MCT1, further suppressing T-cell function (164). Combining ICI therapy with COX 2 or MCT1 inhibition, or neutrophil-focused PD-L1 blockade, may unlock neutrophils, restoring both neutrophil and T-cell antitumor activity.

Redirecting polarization toward an N1 antitumor program offers another path forward. N1 features are supported by robust IFN signaling, whereas TGF-β promotes conversion to an N2 protumor state (116). Consequently, TGF-β inhibition can enable neutrophil-mediated tumor control. In HCC, low CXCL9 expression frequently coincides with impaired N1 polarization and weaker checkpoint responses (165, 166). Strategies include strengthening the CXCL9 and IFN-γ circuit, deploying small molecules that favor N1 skewing, or engineering neutrophils ex vivo for reinfusion to cooperate with ICIs.

From the perspective of physical barriers, neutrophil elastase contributes to extracellular matrix remodeling and fibrosis in the cirrhosis HCC setting, erecting biochemical and structural obstacles to lymphocyte trafficking (167). Pairing neutrophil-directed therapies with antifibrotic or matrix-modifying approaches, such as TGF-β inhibitors or matrix metalloproteinase modulators, may improve T-cell infiltration and amplify checkpoint efficacy. For clinical translation, a composite biomarker framework that incorporates NET-derived plasma markers such as H3Cit-DNA and MPO-DNA (168), the frequencies of suppressive neutrophil subsets including PLAUR^+^ and CD10^+^ALPL^+^ cells (41, 112), and circulating IL-8 and CXCL9 levels could aid stratification and response prediction (165, 169). Radiomic analyses further suggest that computed tomography-based radiomic NET-associated signatures can forecast responses to immunotherapy in patients with HCC (170).

Future clinical trials should consider concurrent or sequential application of neutrophil-targeting therapies with ICIs, such as CXCR2 inhibition, PAD4 inhibition, or COX-2 combination therapy, supplemented by measures like immune profiling and single-cell sequencing analysis, to enhance the efficacy of HCC immunotherapy. In summary, neutrophils represent a novel target for HCC immunotherapy. Their plasticity, diverse involvement mechanisms, and emerging preliminary clinical evidence confer significant potential to this strategy, warranting further exploration in future combination immunotherapy regimens.

## 4 Considerations for HCC associated with liver cirrhosis when applying combined immunotherapy

In patients with HCC and cirrhosis undergoing immune checkpoint inhibitor (ICI) therapy or combination immunotherapy regimens, it is essential to balance oncological benefits against hepatic functional risks (171, 172). The Child-Pugh score (CP) is currently the standard clinical tool for assessing liver function, with most randomized clinical trials requiring Child-Pugh Class A as an inclusion criterion (158). Recent studies have demonstrated that HCC patients with Child-Pugh grade B can still benefit from monotherapy or combination immunotherapy. A meta-analysis by Xie et al. of 699 Child-Pugh B patients demonstrated that some Child-Pugh B patients can achieve objective responses to ICIs, though they exhibit poorer overall survival and outcomes more readily constrained by baseline liver function. This suggests immunotherapy remains a viable option for suitable populations with limited hepatic reserve, rather than an absolute contraindication (173). However, due to the heavier burden of underlying cirrhosis and portal hypertension in Child-Pugh B patients, immune-related adverse events are more likely to lead to hepatic decompensation and portal hypertension complications. For example, in studies of combination immunotherapies such as atezolizumab plus bevacizumab, Child-Pugh B patients experienced higher rates of serious adverse events compared to Child-Pugh A patients, including gastrointestinal bleeding, hyperbilirubinemia, and neutropenia (174). Therefore, when administering combination immunotherapy to such patients, enhanced monitoring of liver function and bleeding risk is essential, and the potential tumor benefits must be individually weighed against the risk of liver function deterioration.

In contrast, existing randomized controlled trials and the vast majority of studies have almost entirely excluded Child–Pugh C patients. In this group, prior systemic therapies (including ICIs and related combination regimens) have not demonstrated benefits in overall survival (OS) or progression-free survival (PFS), leading to their contraindication in this population (175). Concurrently, the AASLD guidelines recommend systemic therapy for Child-Pugh A and select Child-Pugh B patients. For Child-Pugh C patients, supportive care and liver transplantation are primarily recommended, with routine systemic therapy not advised (172).

When considering combination immunotherapy in cirrhotic HCC, several practical precautions are advisable. First, choose combinations with prior evidence of efficacy or tolerable toxicity in populations with compromised liver function, drawing on subgroup and safety data from earlier studies (176). Second, for patients with marginal liver reserve, dose adjustments, extended monitoring intervals, or earlier discontinuation thresholds may help prevent treatment-related hepatic decompensation. Furthermore, enhanced dynamic monitoring of liver function should be implemented preoperatively and during treatment, including parameters such as bilirubin, albumin, INR, and ascites management indicators. Continuous, objective liver function scores like Albumin-bilirubin (ALBI) should be introduced and combined with CP for liver function risk stratification and early detection of deterioration (177). Finally, CP-B patients should routinely receive individualized risk–benefit evaluations, be preferentially considered for clinical trials, and have treatment decisions made within multidisciplinary teams. In short, relying solely on Child–Pugh status is insufficient; a multiparametric, personalized, and closely monitored approach is essential to balance safety and potential therapeutic gain.

## 5 Conclusion and perspectives

**Figure 1:**
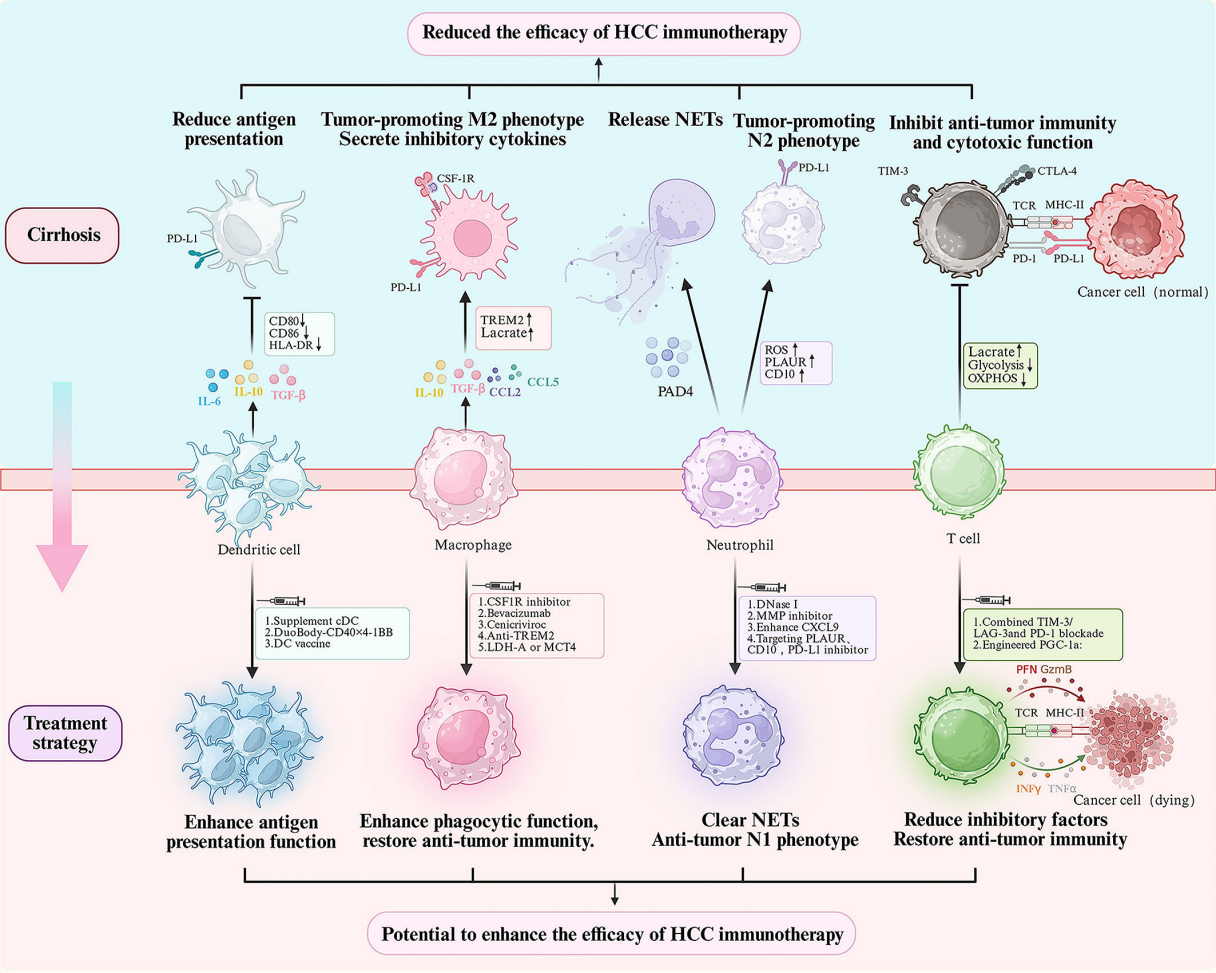
Impact of the cirrhosis-associated immune microenvironment on the efficacy of HCC immunotherapy. Liver cirrhosis induces remodeling of the number and function of key immune cells, including dendritic cells, macrophages, neutrophils, and T cells, thereby establishing an immunosuppressive microenvironment that diminishes HCC’s response to immunotherapy. Specific modulation and functional reprogramming of these cellular states may represent a potential strategy to enhance the efficacy of HCC immunotherapy.

This review maps how cirrhosis reshapes the major immune lineages in the liver—DCs, T lymphocytes, macrophages, and neutrophils—building a coordinated suppressive network characterized by weakened antigen display, intensified cytokine and checkpoint signaling, remodeling of stroma and vasculature, and metabolic fatigue (**Figure 1**). Together these shifts help explain the modest effectiveness of ICI therapy in HCC. In mechanistic terms, cirrhosis reduces DC abundance and maturation, imposes exhaustion and metabolic limits on T cells, skews KCs and scar-associated macrophages toward tolerance-oriented states that drive extracellular matrix accumulation, and expands NETs and inhibitory subsets. The combined effect is a decline in both the frequency and durability of responses under checkpoint blockade.

Important uncertainties remain. Much of the mechanistic literature relies on animal work or *in vitro* systems, which do not fully capture the diversity of human etiologies, slow temporal course, or spectrum of liver function observed in patients with cirrhosis and HCC. Spatial heterogeneity within fibrotic liver and tumors is also underexplored, and conventional single-cell transcriptomics cannot fully resolve location-specific functions. In individuals with impaired or decompensated hepatic reserve, aggressive immune activation or strategies that suppress myeloid cells and target fibrotic pathways can raise the risks of liver toxicity and infection, demanding study designs that balance antitumor efficacy with organ protection. Furthermore, DCs and macrophages can participate in both antifibrotic and antitumor processes, rendering interventions aimed at these compartments context-dependent and sometimes paradoxical.

Immune checkpoint inhibitors (ICIs) can improve the prognosis of HCC, but their efficacy is closely associated with underlying liver disease and the progression of fibrosis. Regarding cirrhosis, strategies such as the previously mentioned CCR2/CCR5 antagonists targeting macrophages (178) or NET inhibition (37) may be combined with immunotherapy to simultaneously reduce fibrosis and suppress HCC progression, offering a reliable therapeutic approach for cirrhosis-associated HCC. For HBV- and HCV-associated HCC, ICIs do not significantly increase viral reactivation under standardized antiviral therapy and monitoring, with hepatotoxicity primarily manifesting as reversible immune-mediated liver injury (179). The probability of ICIs inducing hepatitis reactivation is also relatively low. In one meta-analysis, only 4 out of 878 HCC patients with HBV or HCV experienced HBV reactivation, with no HCV reactivation reported (180). Treatment efficacy in HCC patients with alcoholic liver disease is comparable to that in viral liver disease, with no evidence suggesting ICIs specifically exacerbate alcoholic liver disease (181). Conversely, NASH-associated HCC shows markedly lower survival benefit from ICIs than viral HCC, and may even worsen inflammation, fibrosis, and tumorigenesis (182). Thus, current immunotherapy regimens exhibit dual effects on tumors, with clinical benefits remaining unclear across different etiologies. Outcomes largely depend on the etiology and liver function status. Therefore, based on the combination therapy approaches outlined in the treatment strategies section, comprehensive pre-treatment assessment is essential. Personalized treatment strategies should be adopted, accompanied by rigorous monitoring throughout the entire treatment course.

To interrogate immune–metabolic crosstalk and safety, future work should emphasize models that more closely mirror human disease, including long-duration fibrosis across multiple causes, humanized mice, and organoid platforms. Clinical studies incorporating longitudinal sampling from tumor core and margin in liver tissue, blood, and ascites, together with integrated profiling of single-cell, spatial, and metabolomic layers, will facilitate the reconstruction of cellular circuits and enable identification of early intervention nodes. In parallel, pragmatic biomarkers deserve development and validation, including NET-related markers in plasma such as H3Cit-DNA and MPO-DNA, neutrophil subsets marked by PLAUR or CD10 with ALPL, and chemoattractants such as IL-8 and CXCL9 for stratification and prediction. Early-phase trials are needed to test combinations that pair immune cell-targeted agents with ICI therapy. These combinations include the following: PAD4 inhibitors plus checkpoint blockade in cohorts with high NET signatures or enriched PLAUR^+^ neutrophils; anti-TREM2 or CSF1R inhibitors plus ICI therapy in patients with TREM2^+^ or scar-associated macrophage-rich tumors; and CXCR1 or CXCR2 inhibitors plus ICI therapy in settings with CXCR2 activation or elevated IL-8. Continued exploration of antifibrotic and immunotherapy combinations is warranted, with stringent monitoring for hepatic toxicity, infection, and procedure-related complications.

In summary, cirrhosis is not a mere backdrop for HCC, but a central driver of immunotherapy outcomes. Only through mechanism-guided, patient-centered, incorporating longitudinal sampling across disease progression, supported by rigorous preclinical validation and thoughtful integration of cirrhosis-related biomarkers, can the concept of relieving fibrosis-associated immunosuppression be translated into a safe and workable clinical strategy for affected patients.

## Glossary

ACLF: Acute-on-chronic liver failure
AD: Acute decompensated cirrhosis
AhR: Aryl hydrocarbon receptor
ALBI: Albumin-bilirubin
Ang-2: Angiopoietin-2
CAFs: Cancer-associated fibroblasts
CAR-T: Chimeric antigen receptor T-cell
CCL: C-C motif chemokine ligand
CCR: C-C chemokine receptor
cDC: Classical dendritic cells
cGAS: Cyclic GMP-AMP synthase
COX2: Cyclooxygenase 2
CP: Child-Pugh
CSF 1: Colony-stimulating factor 1
CTLA-4: Cytotoxic T-lymphocyte-associated protein 4
CXCL: C-X-C motif chemokine ligand
DAMPs: Damage-associated molecular patterns
DCs: Dendritic cells
DNase: Deoxyribonuclease
FAO: Fatty-acid oxidation
FLDCs: Fibrotic liver dendritic cells
G-CSF: Granulocyte colony stimulating factor
GzmB: Granzyme B
HCC: Hepatocellular carcinoma
HDAC: Histone deacetylase
HLA: Human leukocyte antigen
HMGB1: High mobility group box 1
HSCs: Hepatic stellate cells
ICIs: Immune checkpoint inhibitors
IDO1: Indoleamine 2,3-dioxygenase 1
IFN-γ: Interferon-γ
IRF4: Interferon regulatory factor 4
JAK: Janus kinase
KCs: Kupffer cells
KP: Kynurenine pathway
Kyn: Kynurenine
LAG-3: Lymphocyte activation gene 3
LDH-A: Lactate dehydrogenase A
LPS: Lipopolysaccharide
MCD: Methionine- and choline-deficient diet
MCT: Monocarboxylate transporter
MDSCs: Myeloid-derived suppressor cells
MMP: Matrix metalloproteinases
moDCs: Monocyte-derived DCs
MoMφs: Monocyte-derived macrophages
MPO: Myeloperoxidase
mTOR: Mechanistic target of rapamycin
NE: Neutrophil elastase
NETs: Neutrophil extracellular traps
NF-κB: Nuclear factor-κB
NK: Natural killer
NLDCs: Normal liver dendritic cells
OXPHOS: Oxidative phosphorylation
PAD4: Peptidylarginine deiminase 4
PD-1: Programmed cell death protein 1
pDC: Plasmacytoid dendritic cells
PD-L1: Programmed death ligand 1
PI3K: Phosphoinositide 3-kinase
PLAUR: Plasminogen activator urokinase receptor
PPAR: Peroxisome proliferator-activated receptor
ROS: Reactive oxygen species
SC: Stable cirrhosis
SIRPα: Signal regulatory protein alpha
SLC7A11: Solute carrier family 7 member 11
STAT: Signal transducer and activator of transcription
STING: Stimulator of interferon genes
TACE: Transarterial chemoembolization
TAMs: Tumor-associated macrophages
TANs: Tumor-associated neutrophils
TGF-β: Transforming growth factor-β
TIGIT: T-cell immunoreceptor with Ig and ITIM domains
TIM-3: T-cell immunoglobulin and mucin domain-containing 3
TLR: Toll-like receptor
TNF-α: Tumor necrosis factor-α
TOX: Thymocyte selection-associated high mobility group box
Treg: Regulatory T cell
TREM2: Triggering receptor expressed on myeloid cells 2
VEGF: Vascular endothelial growth factor.
